# Novel typing of iliac vein compression in asymptomatic individuals evaluated by contrast enhanced CT

**DOI:** 10.1007/s00276-021-02678-w

**Published:** 2021-01-22

**Authors:** Jiaying Li, Haibo Chen, Wujie Chen, Kefeng Zhou, Zhichao Xu, Maosheng Xu, Zhichao Sun

**Affiliations:** 1 The First Clinical Medical College of Zhejiang Chinese Medical University, Hangzhou, 310053 China; 2 Department of Radiology, The First Affiliated Hospital of Zhejiang Chinese Medical University, 54 Youdian Road, Hangzhou, 310006 China; 3 Department of Radiology, The Cancer Hospital of the University of Chinese Academy of Sciences (Zhejiang Cancer Hospital), Hangzhou, 310022 China

**Keywords:** Iliac vein, Compression type, Asymptomatic diseases, May-Thurner syndrome, Iliac vein compression syndrome, Tomography, X-ray computed

## Abstract

**Purpose:**

Compression of the iliac vein between the iliac artery and lumbosacral vertebra can cause iliac vein compression syndrome (IVCS). The purpose of this study is to assess compression characteristics and establish a new sub-typing in asymptomatic IVCS individuals using contrast-enhanced CT.

**Methods:**

A retrospective analysis of abdomen contrast-enhanced CT images from 195 asymptomatic subjects with iliac vein compressed was investigated. Patients had no history of venous pathology, and images were collected from June 2018 to January 2019. Qualitative and quantitative characteristics of compression were examined including the location, pattern, minor diameter, area, and the percentage compression on an orthogonal section by the post-processing of multiple planar reconstruction and volume rendering.

**Results:**

There were 107 females and 88 males with age range 18–92 years. The most common site of iliac vein compression was localized to the left common iliac vein (LCIV) (178/195, 91.3%). Notably, four compression types (type I–IV) were established according to the compression location, with type II being the most common. The four compression types had differences in the upper limit and fluctuation range of compression. It was found that the average level of iliac vein compression was below 25%. The compression degree of the left common iliac vein in type II was relatively concentrated, and the upper limit of compression was close to 70%.

**Conclusion:**

Asymptomatic iliac vein compression was categorized according to compression location. The proposal of four types might help clinicians to predict which IVCS patients would benefit from interventional therapy.

## Introduction

Compression of the iliac vein between the iliac artery and lumbosacral vertebra causes deep vein thrombosis or venous hypertension in the lower extremity, known as the iliac vein compression syndrome (IVCS) or May-Thurner syndrome (MTS) or Cockett syndrome [2, 7, 12]. It is categorized with three clinical stages divided by the severity of symptoms; stage I, asymptomatic iliac vein compression; stage II, development of intraluminal fibrous band; stage III, occurrence of deep vein thrombosis [14]. This venous obstructive lesion not only correlates closely to extrinsic compression between the overlying iliac artery and the vertebra but also involves hemodynamics.

In asymptomatic stage, many subjects were found to have compression of iliac vein in CT examination without the establishment a collateral pathway or venous-related diseases in the leg, but it may have an effect on the venous reflux. These asymptomatic individuals with long durations of severe compression or previous vascular trauma will often progress into the symptomatic stage [3, 5, 22]. The classic location of compression is typically in the left common iliac vein (LCIV) by the right common iliac artery (RCIA), which can be displayed by computed tomography. Multi-slice spiral CT enables offer robust spatial resolution due to the post-processing capabilities of multiple planar reformation (MPR), curved planar reformation (CPR), and volume rendering (VR) [11]. Furthermore, the use of multi-planar reformation (MPR) allows the formation of 2-dimensional images and 3-dimensional volume rendering (VR) to render a detailed image, which enables a greater assessment of iliac vein compression.

However, the patterns of asymptomatic iliac vein compression were not reported as the majority of studies focused on IVCS in case reports or unilateral vein compression [1, 4, 19, 21]. Moreover, the cutoff value of for the diagnosis of IVCS from asymptomatic to symptomatic compression remains controversial. Therefore, the purpose of this study was to observe, evaluate and summarize the compression characteristics and degree of stenosis of bilateral iliac vein in asymptomatic individuals without lower extremity diseases by contrast-enhanced CT, and to establish a subdivided compression typing to assist and optimize clinical decision-making.

## Materials and methods

### Subjects

This retrospective study was approved by the hospital institutional review board and the informed consent requirement was waived. Medical records and abdominal contrast-enhanced CT scans were conducted in 598 consecutive subjects from June 2018 to January 2019.

Inclusion criteria: (1) age ≥ 18 years; (2) contrast-enhanced CT of the lower abdomen or whole abdomen performed; (3) without lower extremity vascular-related symptoms and signs (edema, swelling, pain, skin pigmentation, venous ulcer). Exclusion criteria included: (1) poor quality of images; (2) percentage compression of the minor diameter less than 5%; (3) intra-abdominal lesion that caused extrinsic compression to iliac vein (occupying lesion, aneurysm, hematoma); (4) presence of the collateral pathways around the compression; (5) history of lower extremity vascular-related disease (varicose veins, DVT, thrombophlebitis) and history of trauma, abdominal surgery or vascular bypass surgery.

In all, 195 asymptomatic subjects were enrolled in our study including 107 females and 88 males. The mean age was 60.0 years (range 18–92 years).

### Image acquisition

All images were obtained with an Aquilion ONE (Toshiba Medical Systems, Tokyo, Japan) CT scanner. The main scanning parameters were as follows: tube voltage, 120 kVp; automatic tube current 100–250 mAs; collimation 0.5 mm × 80; matrix 512 × 512; reconstruction slice thickness, 1.0 mm; reconstruction interval, 0.8 mm; rack rotation speed 0.35 r/s; reconstruction soft tissue algorithm. Contrast material (Iodixanol 320 [milligrams of iron per milliliter], 1 mL/kg) was injected intravenously (at 3 mL/s). Patients underwent dual-phase (arterial, after 25–35 s; venous, after 25–35 additional seconds) acquisitions with a reconstruction slice thickness of 1.0 mm.

All image measurements were conducted with orthogonal reconstructions on GE AW 2.0 Workstations (GE HealthCare, USA), where digitally enlarged images and calibrated measurement tools by three-dimensional (3D) images reconstructed by MPR, CPR and VR.

### Data measurement and observation

The orthogonal section was defined as the perpendicular line to the long axis of the iliac vein at the intersection of the iliac artery. The MPR and VR methods were used to analyze and measure data.

**Fig. 1:**
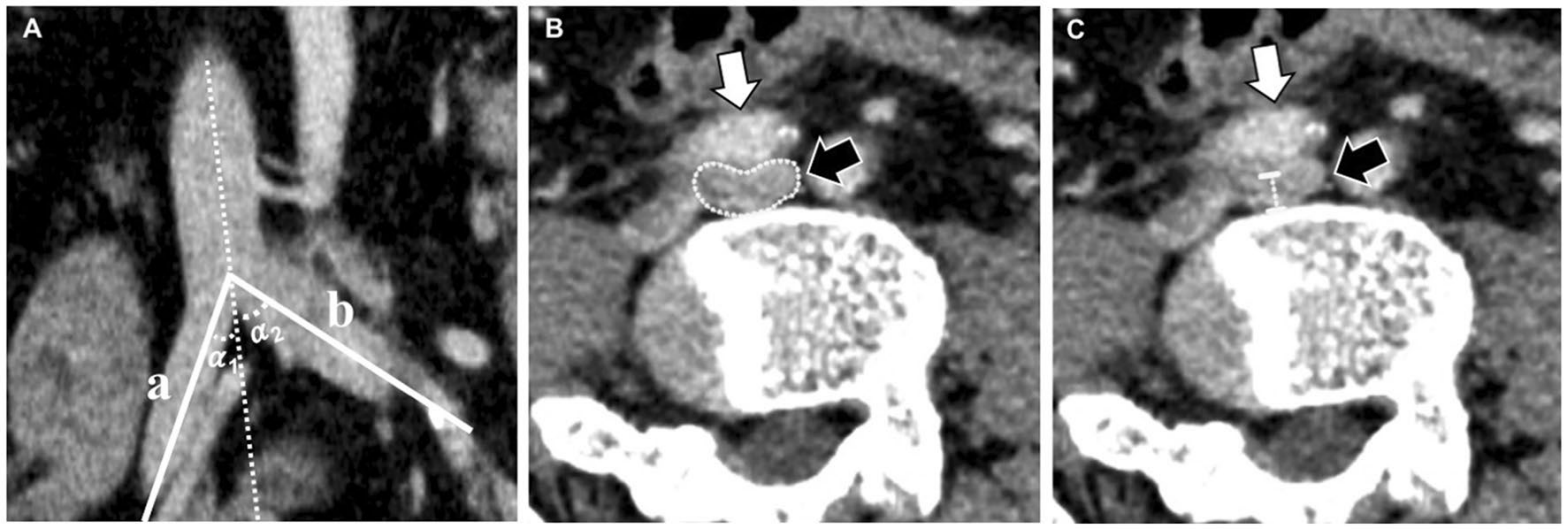
Measurement on an orthogonal section by multiple planar reconstruction. **a** The angle (*α*_1_, *α*_2_) between the right (**a**) or left (**b**) common iliac vein and inferior vena cava at confluence level of the common iliac vein; **b, c** the minor diameter (**b**) and area (**c**) at the site of the maximum iliac vein (black arrow) compression by the iliac artery (white arrow)

Quantitative measures included the angle between common iliac vein (CIV) and inferior vena cava (IVC) at the confluence level of the CIV, the minor diameter and area at the site of maximal iliac vein compression by the iliac artery, the area proximal (1 cm) and distal (1 cm) to the site of maximal iliac vein compression, compression range, and percentage compression. The percentage compression (compression degree) was calculated by dividing the minor area of the iliac vein at maximal compression by the mean area of the uncompressed proximal (1 cm) and distal (1 cm) iliac vein (Fig. 1). Qualitative observations included the location of maximal compression, the pattern of compression, and spinal level at which the maximum compression occurred, which were composed of the compression of the left- and right-sided iliac veins. Finally, four common compression types were summarized and established.

All data were measured on the highest-quality image. The projection and orientation were adjusted arbitrarily to match the anatomical structures, such as the anterior CIA, middle CIV, and posterior lumbosacral vertebral. Compression was defined as a deformation of the CIV on the orthogonal section. Two experienced, board-certified radiologists **(**JY L with 8-year experience in CT abdominal diagnosis and HB C with 15-year experience in CT abdominal diagnosis and intervention therapy) independently read and analyzed all images. The two groups of interpretation were blind to each other. Any discrepancies in the interpretation were resolved by consensus at subsequent meetings.

### Statistical analysis

All statistical analyses were performed using SPSS version 25.0 statistical package (IBM, Chicago, IL, USA). Continuous variables with the normal distribution were described as mean ± standard deviation (SD) or median (interquartile range [IQR]) with the skewed distribution, and categorical variables as frequency (percentage). A qualitative estimate of iliac vein compression was estimated by rating the level of CIV compression (≤ 25%, 25%–50%, ≥ 50%), by age (< 60 years, ≥ 60 years), by gender (female, male), and four compression types (type I, type II, type III, type IV). Statistical significance was tested using Student’s *t* test or One-way ANOVA test for continuous normally distributed variables, and Mann–Whitney *U* test or Kruskal–Wallis *H* test used for an abnormally distribution. Pearson’s Chi‑square test or Fisher’s exact test was used to compare differences in proportion between groups. A *p* value < 0.05 was considered statistically significant. The intra-observer agreement for indices was evaluated with the intra-class correlation coefficient (ICC). An ICC was greater than 0.75 was considered in good agreement.

## Results

This study evaluated 107 females (107/195, 54.9%) and 88 males (88/195, 45.1%) who were asymptomatic. The mean age was 60.0 years of age (range 18–92 years). The intra-observer ICC calculated based on the two measurements ranged from 0.8 to 0.9 and indicated favorable intra-observer reproducibility.

### General iliac vein compression characteristics and compression sites

The site of iliac vein compression was localized to the left common iliac vein (LCIV, 178/195, 91.3%), right common iliac vein (RCIV, 54/195, 27.7%), confluence of the inferior vena cava (CIVC, 10/195, 5.1%), left external iliac vein (LEIV, 6/195, 3.1%), confluence of the left common iliac vein (CLCIV, 1/195, 0.5%), and the right external iliac vein (REIV, 1/195, 0.5%). The compression of the LCIV had the highest incidence (*χ*^2^ = 414.88, *p* < 0.001).

In Table 1, it was shown that the minor diameter of the LCIV was smaller than that of the RCIV (6.1 ± 2.6 mm, 8.3 ± 2.8 mm, respectively; *p* < 0.001) and the compression range of the LCIV was larger than the RCIV (11.2 ± 2.1 mm, 9.9 ± 2.3 mm, respectively; *p* < 0.001). There was no statistically significant difference between the median percentage compression of the LCIV and the RCIV [22.4 (28.1) %, 22.1 (20.3) %, respectively] and percentage compression categories between the LCIV and the RCIV (*p* > 0.05). The left-sided angle between the common iliac vein (CIV) and the inferior vena cava (IVC) at the confluence level of the CIV was found to be significantly larger than that of the right-sided angle (*p* < 0.001).

**Table 1:** Compression characteristics of LCIV and RCIV

Characteristics	LCIV (*n* = 178)	RCIV (*n* = 54)	Statistics	*p*
Age (years)	60.0 ± 12.4	61.2 ± 10.6	0.62^§^	0.54
Gender				
Male	80 (44.9%)	29 (53.7%)	1.28^‡^	0.26
Female	98 (55.1%)	25 (46.3%)		
Minor diameter (mm)	6.1 ± 2.6	8.3 ± 2.8	5.50^§^	< 0.001***
Minor area (mm^2^)	111.6 ± 47.8	117.9 ± 42.6	0.87^§^	0.39
Compression range (mm)	11.2 ± 2.1	9.9 ± 2.3	3.75^§^	< 0.001***
Percentage compression (%)				
Median (IQR)	22.4 (28.1)	22.1 (20.3)	0.55^†^	0.58
≤ 25%	99 (55.6%)	33 (61.1%)	0.92^‡^	0.63
25–50%	63 (35.4%)	18 (33.3%)		
≥ 50%	16 (9.0%)	3 (5.6%)		
Angle between CIV and IVC at confluence level of CIV (^o^)				
Left-sided	39.3 (20.1)	35.8 (15.8)	0.44^†^	0.66
Right-sided	18.8 (10.7)	17.7 (9.8)	0.08^†^	0.94
Statistics	13.40^†^	7.88^†^		
*p*	< 0.001***	< 0.001***		

Data are presented as mean ± SD, Median (IQR), or *n* (%)

*SD* standard deviation, *IQR* interquartile range, *CIV* common iliac vein, *IVC* inferior vena cava, *LCIV* left common iliac vein, *RCIV* right common iliac vein

***p < 0.001

^§^*t* Value

^‡^*χ*^2^ value

^†^*U* value

Data describing the compression site of the LEIV were obtained from 3 males and 3 females with an age of 58.5 ± 4.3 years, a minor diameter of 5.5 ± 1.9 mm, a minor area of 101.8 ± 18.4 mm^2^, a compression range of 11.8 ± 1.6 mm and a median percentage compression of 33.2 (10.3) %. Meanwhile, compression of REIV was obtained from a single, 58-year-old male with a minor diameter of 3.8 mm and a minor area of 52.9 mm^2^. The compression range was 19 mm and the percentage compression was 41.48%. The quantitative data of compression site at the CIVC or CLCIV were not measured.

### Comparison of minor area and percentage compression of LCIV and RCIV

Men and women were further separated by age (< 60 years and ≥ 60 years). Significant differences in the minor area of the LCIV between both genders and ages were found (*p* < 0.01). The percent compression of the LCIV in women was significantly different between those over and under 60 years of age (*p* < 0.05). The minor area of the RCIV was also significantly affected by gender in those over 60 years (*p* < 0.05). No significant differences in the percent compression of the LCIV or RCIV were found between genders (Tables 2 and 3).

**Table 2:** Comparison of the minor area (mm^2^) of LCIV/RCIV

	LCIV (*n* = 178)		Statistics	*p*	RCIV (*n* = 54)		Statistics	*p*
	< 60 years (*n* = 82)	≥ 60 years (*n* = 96)			< 60 years (*n* = 24)	≥ 60 years (*n* = 30)		
Male	125.2 ± 46.6	131.5 ± 52.8	0.57^§^	0.57	134.9 ± 49.8	130.1 ± 44.1	0.28^§^	0.78
Female	97.0 ± 40.3	98.3 ± 42.5	0.16^§^	0.88	108.5 ± 30.2	95.1 ± 33.9	1.04^§^	0.31
Statistics	2.93^§^	3.42^§^			1.58^§^	2.38^§^		
*p*	0.004**	0.001**			0.13	0.03*		

Data are presented as mean ± SD

*SD* standard deviation, *LCIV* left common iliac vein, *RCIV* right common iliac vein

*p < 0.05

**p < 0.01

^§^*t* Value

**Table 3:** Comparison of percentage compression (%) of LCIV/RCIV

	LCIV (*n* = 178)		Statistics	*p*	RCIV (*n* = 54)		Statistics	*p*
	< 60 years (*n* = 82)	≥ 60 years (*n* = 96)			< 60 years (*n* = 24)	≥ 60 years (*n* = 30)		
Male	22.4 (24.8)	20.4 (25.2)	0.73^†^	0.47	20.1 (18.4)	20.7 (18.4)	0.71^†^	0.48
Female	27.8 (26.0)	17.0 (31.0)	2.15^†^	0.03^*^	22.3 (22.0)	33.0 (36.5)	1.14^†^	0.25
Statistics	0.62^†^	1.11^†^			0.81^†^	1.15^†^		
*p*	0.54	0.27			0.42	0.25		

Data are presented as median (IQR)

*IQR* interquartile range, *LCIV* left common iliac vein, *RCIV* right common iliac vein

^†^*U* value

By grading the level of CIV compression (≤ 25%, 25–50%, ≥ 50%), 35.4% of patients demonstrated a compression degree between 25 and 50% at the LCIV and 9% had a compression degree greater than 50%. Similarly, 33.3% had a compression degree between 25 and 50% at the RCIV and 5.6% had a compression degree greater than 50%. No significant differences were found between genders. Patients with a 25–50% compression of the LCIV and RCIV accounted for 35.4% and 33.3% of cases, respectively (Table 4).

**Table 4:** Comparison of percentage grading of LCIV/RCIV

	LCIV (*n* = 178)		Total	RCIV (*n* = 54)		Total
	Male	Female		Male	Female	
≤ 25%	46 (57.5%)	53 (54.1%)	99 (55.6%)	21 (72.4%)	12 (48.0%)	33 (61.1%)
25–50%	29 (36.3%)	34 (34.7%)	63 (35.4%)	7 (24.1%)	11 (44.0%)	18 (33.3%)
≥ 50%	5 (6.2%)	11 (11.2%)	16 (9.0%)	1 (3.5%)	2 (8.0%)	3 (5.6%)
Statistics	1.34^‡^			3.43^‡^		
*p*	0.51			0.18		

Data are presented as *n* (%)

*n* Number, *LCIV* left common iliac vein, *RCIV* right common iliac vein

^‡^*χ*^2^ value

**Fig. 2:**
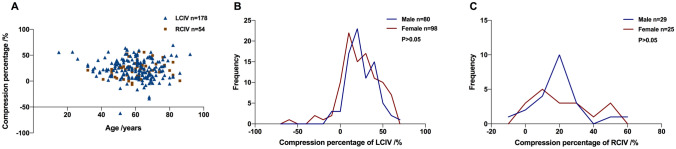
The compression percentage of LCIV and RCIV by age and gender. **a** The percentage compression of LCIV and RCIV by age. **b, c** The frequency distribution of LCIV (**b**) and RCIV (**c**) percentage compression in females and males. *LCIV* left common iliac vein, *RCIV* right common iliac vein

The compression percentage of LCIV and RCIV by age and gender was shown in Fig. 2a–c. The percentage compression of LCIV in males and females was 22.1 (24.7) % and 23.1 (29.9) %, respectively (*U* = 0.38, *p* > 0.05), that of RCIV in males and females was 20.7 (16.1) % and 25.9 (35.9) %, respectively (*U* = 1.17, *p* > 0.05).

### General compression characteristics of iliac vein compression types

**Fig. 3:**
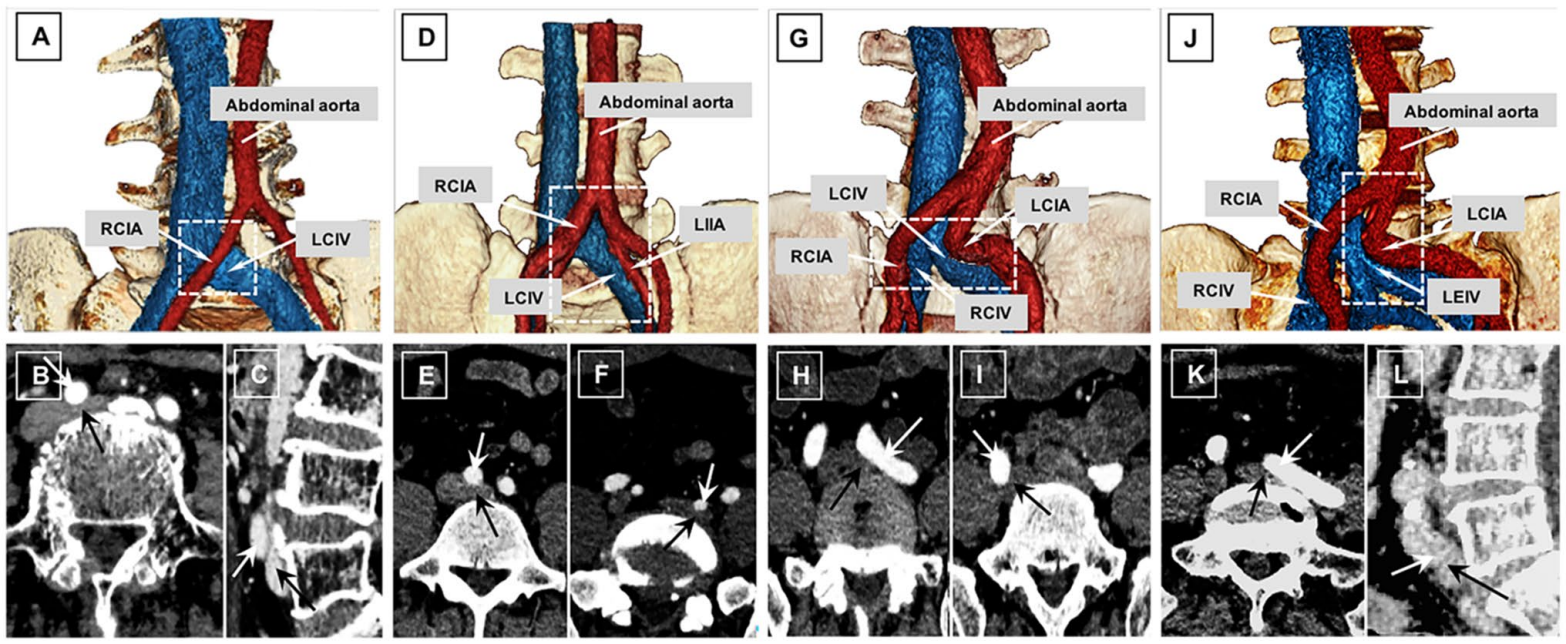
A diagram of the four compression types in a volume rendering (**a**, **d**, **g**, **j**) and contrast-enhanced CT (axial, arterial phase, **b**, **e**, **f**, **h**, **i**, **k**; and sagittal reformation at venous phase, **c**, **l**). Type I** a–c** sole compression of LCIV (**a**, dotted square), the LCIV (black arrow) compressed by RCIA (white arrow); Type II** d–f** dual compression of the LCIV (**d**, dotted square), the LCIV (**e**–**f**, black arrow) compressed by RCIA (**e**, white arrow) and LIIA (**f**, white arrow) simultaneously; Type III** g–i** bilateral compression of the LCIV and the RCIV (**g**, dotted square), which was the LCIV (**h**, black arrow) compressed by LCIA (**h**, white arrow) and RCIV (**i**, black arrow) compressed by the RCIA (**i**, white arrow); Type IV** j–l** other compression types (**j**, dotted square), such as the LEIV (black arrow) compressed by LCIA (white arrow). *LCIA* left common iliac artery, *LCIV* left common iliac vein, *LEIV* left external iliac vein, *LIIA* left internal iliac artery, *RCIA* right common iliac artery, *RCIV* right common iliac vein

Iliac vein compression was divided into four types: Types I (21.0%, 41/195), II (36.4%, 71/195), III (24.1%, 47/195), and IV (18.5%, 36/195) (Fig. 3). Type I was described as sole compression of the LCIV (e.g. compression of the LCIV by the RCIA, Fig. 3a). Type II was described as dual compression of the LCIV (e.g. compression of the LCIV by the RCIA and LIIA, Fig. 3d). Type III was described as bilateral compression of the LCIV and RCIV (e.g. compression of the LCIV and RCIV by the LCIA and RCIA, Fig. 3g). Type IV was described as other compression types (e.g. compression of the LEIV by the LCIA, Fig. 3j).

**Fig. 4:**
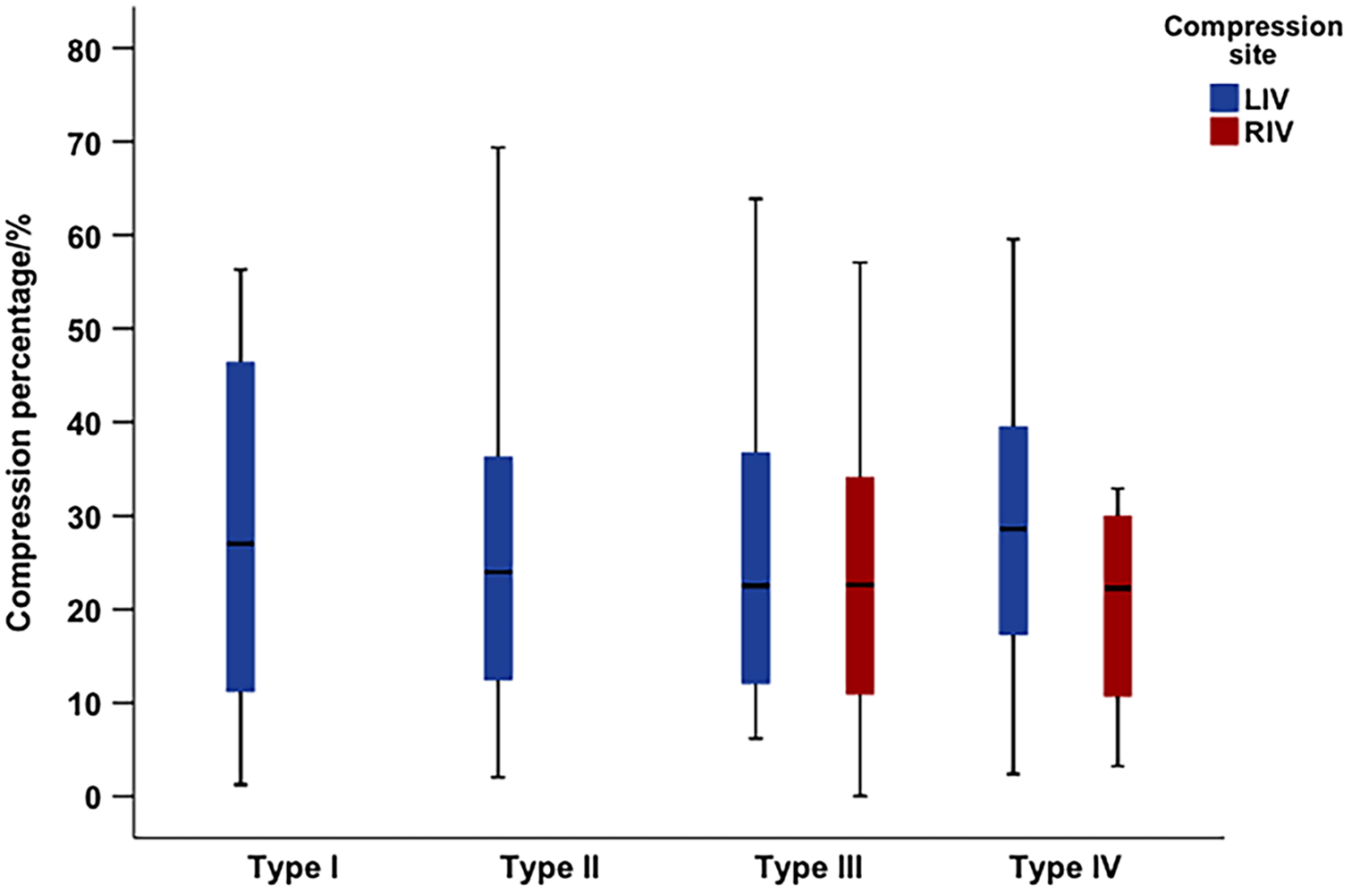
The box-plot of percentage compression above zero percent in four types. *LIV* left iliac vein, *RIV* right iliac vein

The percentage compression of the iliac vein between the four subtypes was further studied as evidenced by the box-plot. It was determined that the average degree of the iliac vein compression in each type was similar. When the bilateral iliac veins were compressed, the compression degree of the left iliac vein was slightly higher than that of the right iliac vein. The compression degree of type I displayed as a single compression of the LCIV was relatively dispersed, and its upper limit was close to 60%. Type II displayed as double compression of the LCIV was relatively concentrated, and the upper limit of compression was close to 70%. The upper limit of compression of right iliac vein in type IV was less than 50%, while the upper limit of compression of the right-sided compression in type III was close to 60% (Fig. 4).

No significant difference in compression type was found either between age, gender, the angle between the CIV at the confluence level of the CIV or percent compression. It was found that 6.1% (12/195), 15.9% (31/195), 27.2% (53/195), 43.1% (84/195), and 7.7% (15/195) of compressions were at L4, L4/L5, L5, L4-L5, and L4/L5-S1, respectively. No spondylolisthesis was observed (Table 5).

**Table 5:** Characteristics of iliac vein compression type

Characteristics	Type I (*n* = 41)	Type II (*n* = 71)	Type III (*n* = 47)	Type IV (*n* = 36)	Statistics	*p*
Age (years)						
Mean ± SD	61.7 ± 13.5	59.2 ± 13.1	60.6 ± 10.7	58.7 ± 12.8	0.52^&^	0.67
< 60 years	15 (16.9%)	35 (39.3%)	22 (24.7%)	17 (19.1%)	1.80^‡^	0.62
≥ 60 years	26 (24.5%)	36 (34.0%)	25 (23.6%)	19 (17.9%)		
Gender						
Male	19 (21.6%)	31 (35.2%)	24 (27.3%)	14 (15.9%)	1.32^‡^	0.72
Female	22 (20.6%)	40 (37.4%)	23 (21.5%)	22 (20.6%)		
Percentage compression (%)						
Median (IQR)	22.6 (37.3)	22.5 (28.1)	21.7 (24.8)	24.2 (23.3)	0.86^††^	0.84
≤ 25%	21 (15.7%)	39 (29.1%)	55 (41.0%)	19 (14.2%)	4.31^‡^	0.64
25–50%	14 (16.3%)	26 (30.2%)	32 (37.2%)	14 (16.3%)		
≥ 50%	6 (31.6%)	6 (31.6%)	4 (21.1%)	3 (15.8%)		
Angle between CIV and IVC at confluence level of CIV (^o^**)**						
Left-sided	42.5 (26.3)	41.9 (20.7)	34.7 (18.1)	35.3 (19.9)	4.70^††^	0.20
Right-sided	16.1 (13.2)	18.8 (9.4)	18.8 (9.9)	16.7 (8.2)	3.03^††^	0.39
Level of spine at iliac vein compression						
L4	4 (9.7%)	4 (5.6%)	2 (4.3%)	2 (5.6%)	45.44^‡^	< 0.001**^*^
L4/L5	15 (36.6%)	7 (9.9%)	4 (8.5%)	5 (13.9%)		
L5	17 (41.5%)	24 (33.8%)	5 (10.6%)	7 (19.4%)		
L4–L5	3 (7.3%)	32 (45.1%)	30 (63.8%)	19 (52.8%)		
L4/L5–S1	2 (4.9%)	4 (5.6%)	6 (12.8%)	3 (8.3%)		

Data are presented as mean ± SD, median (IQR), or *n* (%)

*SD* standard deviation, *IQR* interquartile range, *n* number, *CIV* common iliac vein, *IVC* inferior vena cava, *IVD* intervertebral disc, *L* lumbar vertebra, *S* sacral vertebra, *L4/L5* the intervertebral disc between L4 and L5, *L5/S1* the intervertebral disc between L5 and S1, *L4–L5* L4, L4/L5 and L5, *L4/L5–S1* L4/L5, L5, L5/S1 and S1

***p < 0.001; and *F* value

^‡^*χ*^2^ value

^††^*H* value

## Discussion

Compression of the iliac vein by the anterior iliac artery and posterior lumbosacral vertebra can cause the formation of an intraluminal fibrous band, which can significantly block the return of venous blood and hamper the venous function after repeated and chronic pulsation of the overlying artery. CT is a widely available and non-invasively enables evaluation of entire iliac vein and artery, its branches, and the surrounding organs. The use of contrast-enhanced CT allows the visualization of venous compression, thrombosis, and collateral vessels. Using contrast-enhanced CT, the characteristics of iliac vein compression were evaluated and compression typing was established.

The site of iliac vein compression in asymptomatic subjects was located to the bilateral iliac vein and its branches. The compression location of LCIV and RCIV which was caused by the RCIA, LCIA or branches of the iliac artery were the mostly reported in anatomical variants of the IVCS patients [1, 4, 19, 21]. In this study, we found similar results that the most common site of the compression was the LCIV (91.3%), followed by the RCIV (27.7%), CIVC (5.1%), LEIV (3.1%), CLCIV (0.5%), and REIV (0.5%) in asymptomatic subjects. Furthermore, the angle between CIV and IVC on the left-sided was larger than the right-sided angle, which contributed to the increased prevalence of LCIV compression. Therefore, the different iliac vein compression locations might attribute to the angle between the CIV and IVC.

In addition, four compression types (type I–IV) were established according to compression location, with type II being the most common. Type I was described sole compression of the LCIV, type II described dual compression of the LCIV, type III described bilateral compression of the LCIV and RCIV, type IV described other compression types. Similar to the anatomical classification for aortic dissection, the classification for iliac vein compression considers the location and severity of the defect [16].

In this study, it was also determined that the upper limit and range of variation of compression for the four types of bilateral iliac vein compression were different. According to the classification and degree of iliac vein compression of asymptomatic subjects in this study, it was found that the average level of iliac vein compression was below 25%. The upper limit (50–70%) of left iliac vein compression was higher than the upper limit (30–60%) of right iliac vein compression. When bilateral iliac veins were compressed, the left-sided iliac vein compression degree was slightly higher than the right-sided iliac vein.

This study found that the majority of patients demonstrated compressions between 25 and 50%. Patients with a 25–50% compression of the LCIV and RCIV accounted for 35.4% and 33.3% of cases, respectively, while those with a ≥ 50% compression of the LCIV and RCIV accounted for 9.0% and 5.6% of cases, respectively. These results mirrored the previous study of asymptomatic subjects with LCIV compression [6]. However, in IVCS patients, Narayan et al. [13] showed that 74.8% of subjects had greater than 25% compression and 33.3% had a greater than 50% compression. It indicated that the severe compression was closely related to develop into symptomatic IVCS, but the diagnosis of IVCS should be made by combining the percentage compression with other risk factors for venous thromboembolism, such as a hypercoagulable state, endothelial damage and stasis [3, 22].

Other findings in our study showed that females over the age of 60 had a greater severity of compression. But no such difference was found in males regardless of age. This difference was likely the result of a larger average vessel diameter in males than females [15]. Furthermore, the presence of lumbar lordosis in females and a greater lumbar curvature may have contributed to the findings [9, 17]. Simultaneously, the chosen area to calculate compression percentage may have caused variations from previous studies which used the diameter [6, 13, 15].

In clinical practice, while the critical value of iliac vein compression with symptoms was clinically described as 50% or 70% at present, the value was still controversial [5, 13, 22]. However, there was a lack of clinically individualized assessment of the likelihood of developing symptoms in patients with iliac vein compression. Although endovascular management has evolved and play a significant role in the treatment of IVCS, the endovascular therapeutic techniques and endovascular indications for iliac vein compression syndrome patients were still controversial, such as the stent placement [5, 8, 10, 18, 20]. Therefore, the proposal of this compression classification in this study might assist and optimize clinical decision-making. For example, the stent type and coverage or even preventative placement could be determined according to different compression types individually.

Despite the findings presented herein, several limitations to the existing study are worth noting. As a retrospective study, the small sample size may affect the statistical validity of the reported findings. In addition, the lack of a control group (i.e. symptomatic iliac vein compression) is another factor to consider. The lack of an angiography or intravascular ultrasound was performed as a diagnostic standard. To address these shortcomings, we currently plan to continue this study with an increased sample size and symptomatic IVCS patients as a control.

## Conclusion

The present study determined that iliac vein compression could occur in the bilateral common iliac veins and its associated branches. In the asymptomatic population, iliac vein compression was categorized from type I to IV according to compression location, with type II being the most common. Moreover, the upper limit and fluctuation range of compression could vary in each compression type. Although the average percentage compression of iliac vein was below 25%, the upper limit of left iliac vein compression could reach up to 50–70% and severe compression occurred more commonly in the LIV than in the RIV. The proposal of four compression types in this study which had different the upper limits and fluctuation ranges may help clinicians to predict which IVCS patients will benefit from interventional therapy and optimize clinical decision-making.

## Data Availability

The data that support the findings of this study are available from the corresponding author upon reasonable request.
